# The Time-Course of Sentence Meaning Composition. N400 Effects of the Interaction between Context-Induced and Lexically Stored Affordances

**DOI:** 10.3389/fpsyg.2017.00813

**Published:** 2017-05-26

**Authors:** Erica Cosentino, Giosuè Baggio, Jarmo Kontinen, Markus Werning

**Affiliations:** ^1^Institute of Philosophy II, Ruhr University of BochumBochum, Germany; ^2^Department of Language and Literature, Norwegian University of Science and TechnologyTrondheim, Norway

**Keywords:** affordance, telic component, N400, embodied-simulative account, amodal-symbolic theories, semantic minimalism, truth-conditional pragmatics, compositionality

## Abstract

Contemporary semantic theories can be classified along two dimensions: (i) the way and time-course in which contextual factors influence sentence truth-conditions; and (ii) whether and to what extent comprehension involves sensory, motor and emotional processes. In order to explore this theoretical space, our ERP study investigates the time-course of the interaction between the lexically specified *telic component* of a noun (the function of the object to which the noun refers to, e.g., a funnel is generally used to pour liquids into containers) and an *ad-hoc affordance* contextually induced by the situation described in the discourse. We found that, if preceded by a neutral discourse context, a verb incongruent with the noun's telic component as in “She uses the funnel to *hang* her coat” elicited an enhanced N400 compared to a congruent verb as in “She uses the funnel to *pour* water into a container.” However, if the situation introduced in the preceding discourse induced a new function for the object as an *ad-hoc* affordance (e.g., the funnel is glued to the wall and the agent wants to hang the coat), we observed a crossing-over regarding the direction of the N400 effect: comparing the *ad-hoc* affordance-inducing context with the neutral context, the N400 for the incongruent verb was significantly reduced, whereas the N400 for the congruent verb was significantly enhanced. We explain these results as a consequence of the incorporation of the contextually triggered *ad-hoc* affordance into the meaning of the noun. Combining these results with an analysis of semantic similarity values between test sentences and contexts, we argue that one possibility is that the incorporation of an *ad-hoc* affordance may be explained on the basis of the mental simulation of concurrent motor information.

## Introduction

In this paper we address two relevant dimensions along which contemporary theories of comprehension, of namely the interpretation of words, phrases, and sentences, can be classified. The first refers to the ways and time-course in which contextual factors influence the meanings of sentences. We are particularly interested in the questions how and when a situation introduced in the discourse affects the intuitive truth-conditions of a sentence. The second one regards whether and to what extent comprehension is grounded in sensory, motor and emotional processes. Focusing on the motor domain, we investigate how the affordance of an object in a situation interacts with motor information stored in the lexicon of a noun.

The two sides of the first dimension are Semantic Minimalism (Borg, 2004, 2012) and Truth-conditional Pragmatics (Recanati, 2004). Although both assume the principle of compositionality (Partee, 1984; Werning, 2004, 2005; Pagin and Westerståhl, 2010; Werning et al., 2012), according to which the semantic value of a syntactically complex term is a syntax-dependent function of the semantic values of its syntactic parts, they modify it in two opposite directions. Semantic Minimalism strengthens the principle of compositionality by the assumption of *bottom-up compositionality*, according to which the truth-evaluable semantic content of a sentence is fully determined by its syntactic structure and lexical content where only a small number of lexical items (e.g., indexicals and anaphors) allow for a context-sensitive meaning contribution. With regard to the semantic integration of a sentence in a discourse, this leads to a two-step model: discourse-level information is integrated only after sentence local meaning is established. On the other hand, Truth-conditional Pragmatics weakens the principle of compositionality by the assumption of *free pragmatic enrichment*, which states that contextual information can freely enrich the truth-evaluable content of a sentence at any stage of meaning composition (Recanati, 2004, 2012). Pragmatic enrichment is supposed to be “free” because not only lateral modulations of a word or phrase are allowed, e.g., when the meaning of a word is modulated by the meaning of its argument—*cut the cake* (vertical cutting) vs. *cut the grass* (horizontal cutting)—but any, however remote information can in principle, before sentence meaning composition is completed, modulate the way in which the meaning of a word or phrase contributes to the intuitive truth-conditions of a sentence^1^. Accordingly, a situation introduced in the discourse that precedes the sentence may result in the modulation of the meaning of words or phrases in the sentence before sentence meaning composition is completed. This leads to a single-step model.

As for the second dimension, its two poles are the amodal-symbolic and the embodied-simulative account. Currently most researchers acknowledge that the correct approach lies probably in between these two poles, but the debate is still completely open as to the degree of embodiment and involvement of sensory and motor systems in different processing stages and tasks.

The amodal-symbolic account is based on the conjunction of two theses, that is, that meaning arises from the quasi-syntactic combination of mental symbols (e.g., Fodor, 1975, 2010; Pylyshyn, 1984), and the modularist assumption that meaning is processed in an informationally encapsulated way such that mental symbols are amodal, i.e., largely decoupled from sensory, motor, and emotional processes (Kintsch and Van Dijk, 1978; Kintsch, 1988; McKoon and Ratcliff, 1992; see also Cosentino et al., 2013 for a critical discussion). Given that it is widely agreed in both the amodal-symbolic and the embodied-simulative camps that sensory-motor and emotional activity occurs when a word or a sentence is processed, the debate between the two accounts can be seen as a debate as to whether sensory-motor and emotional processes are constitutive for or just causally related to comprehension (Mahon and Caramazza, 2008). Focusing on the N400 component of the ERP, which has often been related to the core of semantic processing, we hope that we will be able to, at least indirectly, contribute to this debate.

In line with the amodal-symbolic account, Latent Semantic Analysis (LSA; Landauer and Dumais, 1997) has been recently suggested as a computer-linguistic high-dimensional model of meaning similarity and semantic relatedness, based on statistical analyses of patterns of language use in large corpora (Chwilla and Kolk, 2002). LSA crucially assumes that meaning similarity and semantic relatedness of words are fully determined by their relations to other words. Meaning similarity and semantic relatedness thus do not depend on any sensory, motor or emotional processes of speakers and are therefore completely amodal. Evidence in favor of LSA includes studies showing that LSA can be used to retrieve documents that are meaningfully related to queries that do not contain the same words as the documents (Deerwester et al., 1990), grade essays (Landauer et al., 1998), predict coherence judgments (Foltz et al., 1998), and mimic performance of students on the Test of English as a Foreign Language (Landauer and Dumais, 1997). However, against LSA, some authors have argued that the model should be abandoned as it cannot capture the knowledge necessary to predict differences in sensibility judgments between sentences (Glenberg and Robertson, 2000).

The embodied-simulative account claims instead that comprehension is constituted by processes also involved in one's own actions, perceptions and emotions (see, for example, Barsalou, 1999; Gallese and Lakoff, 2005; Prinz, 2005; Kemmerer, 2010; Werning, 2012; Werning et al., 2013). More precisely, comprehension is grounded on the multimodal simulation of perceptions, actions, and emotions (e.g., Barsalou, 1999, 2010; Pecher and Zwaan, 2005; Gibbs, 2006; Glenberg et al., 2013). A distinction needs to be made here between hybrid and full-blooded embodied-simulative accounts. According to many hybrid accounts, little more than the lexical entries, i.e., the primary inputs of semantic composition, are fully and directly grounded in sensory-motor and emotional processes. When it comes to the intermediate and final results of sentence meaning composition, however, these need not be fully embodied, but may as well comprise (quasi-) symbolic structures—e.g., for negation or disjunctions (e.g., Johnson-Laird, 1983, 2006). Full-blooded accounts, in contrast, should maintain that also the outputs of semantic composition are fully and directly grounded in sensory-motor and emotional processes.

Neuroimaging investigations have supported some form of embodied-simulative semantics exploring several different domains. For example, in the domain of perception, it has been shown that perceptual brain regions that process object-related information are also activated by words related to visual features (e.g., “brown”; Pulvermüller and Hauk, 2006), odors (e.g., “cinnamon”; González et al., 2006), sounds (e.g., “telephone”; Kiefer et al., 2008), and taste (“salt”; Barrós-Loscertales et al., 2012). As for actions, it is known that somatotopic areas in the motor and premotor cortex, which are active when subjects move specific body parts (e.g., “face,” “leg,” “arm”), are also active when they understand action-related words that refer to those body parts (e.g., “lick,” “pick,” or “kick”; Pulvermüller, 2005) or comprehend sentences about motion (Tettamanti et al., 2005). Moreover, the semantic processing of action-related verbs is impaired specifically in patients with degenerative brain diseases that affect the motor system, including amyotrophic lateral sclerosis (Grossmann et al., 2008), Parkinson's disease (Cotelli et al., 2007; Boulenger et al., 2008; Rodríguez-Ferreiro et al., 2009), and other motor neuron diseases (Bak et al., 2001). Furthermore, in the domain of emotions, a recent ERP study has found a correlation between empathy measures and the sensitivity to semantic violations regarding emotion words, suggesting that the emotion circuits involved in empathy are also active when subjects process the meaning of emotion-related words (Rak et al., 2013). Recently, the relation between nociceptive processes and the semantic processing of pain-related words has also been explored, showing that when people are presented with pain words, there is substantial activity in the pain matrix, which is also active when people feel a pain (Richter et al., 2010). Consistent with this finding, it has been reported that individual differences in pain sensitivity, as measured by self-report, correlate with people's ratings of the pain-relatedness of words (Reuter et al., 2016). Despite substantial support in favor of the embodied-simulative framework, it should be noted that recent research has provided direct evidence at least against a strong version of embodied semantics, using both psychophysics and neurobehavioral measures (Pavan and Baggio, 2013; Papeo et al., 2015; Ghio et al., 2016).

In the present paper, we want to address both dimensions of the theory space and to this aim we focus on a notion that is particularly relevant in the theoretical framework of the embodied-simulative account, the notion of *affordance*. Following Gibson (1979), affordances are defined as properties things have in virtue of being the object of certain potential actions^2^. The neuroscientific plausibility of this notion is supported by the finding that a set of neurons in the premotor cortex called “canonical neurons” respond not only when manipulable objects are actually manipulated but also when they are simply perceived (Murata et al., 1997 for a study with monkeys; Grèzes and Decety, 2002; Creem-Regehr et al., 2007 for human studies; see for a review Martin, 2007). Moreover, canonical neurons are also active when tool-related nouns are presented (Cattaneo et al., 2010; Marino et al., 2011) and behavioral studies confirm that the processing of nouns can interact with motor activity (Tucker and Ellis, 2004; Lindemann et al., 2006). Furthermore, affordances seem to be involved in the construction of sentence meaning. When people are asked to judge the coherence of two sentences such as “After wading barefoot in the lake, Erik used his shirt to dry his feet” and “After wading barefoot in the lake, Erik used his glasses to dry his feet,” they regard the first sentence as more sensible than the second in spite of the fact that both of them are grammatically well-formed and that the critical words in the sentences, shirt and glasses, are equally unrelated to “dry” as measured by means of LSA (Glenberg and Robertson, 2000).

Here, we introduce a relevant theoretical distinction in the domain of affordances between *ad-hoc affordances* and *generic affordances*. Generic affordances are affordances of a class of objects that are represented as part of the mental concept of that class of objects (e.g., *chair–sit*). *Ad-hoc* affordances are affordances that a particular object has for a particular agent in a particular situation (e.g., *this chair–hide under*, for a child in a peekaboo game). In line with Pustejovsky's Generative Lexicon Theory (1995), generic affordances are often represented as *telic components* in the lexicon of nouns and thus in semantic long-term memory.

The telic lexical components are typically retrieved to understand sentences of the following kind.

1. John began the book (i.e., John began reading the book);
2. John enjoyed the banana (i.e., John enjoyed eating the banana);
3. John used the knife on the turkey (i.e., John used the knife to cut the turkey);
4. John used the funnel for the water (i.e., John used the funnel to pour the water).

In those cases, the telic lexical component fills a certain telic role of the noun that allows to complement the argument of the preceding event-selecting verb (i.e., begin, enjoy, use).

According to Pustejovsky, our knowledge of usual activities associated with objects is encoded by a lexical structure (“Qualia Structure”). The telic component of the lexical entry specifies the function or the purpose of an object. For example, the lexical representation for the artifact noun *funnel* is of the following form:

(1) $$\begin{array}{l} funnel=\lambda x\ldots \exists z\;\exists u\\ \;\left[\begin{array}{l} \ldots \\ \;\left[\begin{array}{cc} \begin{array}{c} F=\\ C= \end{array} & \begin{array}{c} \ldots \\ \ldots \end{array}\\ \begin{array}{c} T=\\ A= \end{array} & \begin{array}{c} (\lambda e)\;\left(pour\left(e,z,u\right)\wedge means\left(e,x\right)\right)\\ \ldots \end{array} \end{array}\right]\\ \ldots \end{array}\right] \end{array}$$

This structure not only specifies the formal (F, the basic category that distinguishes the object within a larger domain, e.g., animate vs. inanimate), the constitutive (C, the relation between an object and its constituent parts), and the agentive (A, factors involved in the object's origin), but also the telic component (T). In the above example, the telic component given in the lexical entry of the word *funnel* specifies that a funnel is used by a human agent (z) as the means to realize the event (e) of pouring something (u).

The lexical structure of a noun allows us to distinguish between verbs that are congruent with the lexical telic component of a noun—called *telically congruent*—and those that are incongruent—called *telically incongruent*. For example, the verb *pour* is telically congruent with *funnel* because it expresses its lexical telic component, whereas the verb *hang* is telically incongruent therewith. It can be assumed that telic congruency and incongruency between the noun and the verb can be quantitatively determined by LSA. A telically congruent noun-verb combination should have higher Semantic Similarity Values (SSVs) than a telically incongruent noun-verb combination.

Linking the two theoretical dimensions mentioned at the beginning, we want to investigate how an *ad-hoc* affordance induced by a preceding discourse interacts with the lexical telic component of a noun. To do so, on the one hand we choose telically congruent and incongruent noun-verb pairs. On the other hand, we contrast a neutral discourse context with a context that induces an *ad-hoc* affordance, which could be expressed by the telically incongruent verb. Given, for example, the telically congruent noun-verb combination *funnel-pour* and the telically incongruent pair *funnel-hang*, the *ad-hoc* affordance-inducing context could specify that the funnel is glued to the wall and the agent has the desire to hang up her coat. In a situation where the *ad-hoc* affordance conflicts with the lexical telic component, a question of priority arises: Is the *ad-hoc* affordance contributing to the intuitive truth-conditions before or after sentence meaning composition is completed? This is where the controversy between Semantic Minimalism and Truth-conditional Pragmatics culminates.

In our study, we address this question focusing on the *N400 component*, a negative deflection in an event-related potential (ERP) waveform peaking around 400 ms after stimulus onset and larger over centro-parietal electrodes (Kutas and Federmeier, 2011). The *N400 effect* is measured as the difference between the amplitudes of the N400 components elicited by two stimuli in different experimental conditions (Baggio and Hagoort, 2011). The N400 component was described for the first time by Kutas and Hillyard (1980) who reported increased amplitude of this component for words whose meanings mismatched with the semantics of the preceding sentence (e.g., “He spread his warm bread with *butter/socks*”). This finding led to the hypothesis that an enhanced N400 component reflects semantic incongruency in language. Later on, additional evidence led to a generalization of this hypothesis, namely that a gradual modulation in the amplitude of the N400 component, measured on a word, corresponds to finer gradations of the expectancy of the stimulus (Kutas and Hillyard, 1984; Kutas et al., 1984). The expectancy of a stimulus depends on several factors, including lexical relations within a sentence. Thus, a semantically unrelated word within a sentence context elicits a larger N400 component as compared to a semantically related word, for instance in the sentence “The girl was writing letters when her friend spilled coffee on the tablecloth/paper” the word “tablecloth” elicits a larger N400 with respect to the word “paper” (Baggio et al., 2008).

There are two main different interpretations as to the functional role of the N400 component. The current experiment is in no way designed to adjudicate between them. However, the two functional interpretations have different impacts on what our experiment actually shows concerning the explored theories of comprehension. Thus, for purely descriptive aims, below we will review the two interpretations of the N400 relating them to the theories' predictions and, in the discussion, to our results. Some accounts of the functional role of the N400 emphasize that it reflects *lexical retrieval processes*. In this view, the amplitude of the N400 component is modulated by the ease of accessing information in semantic memory, which depends on the extent to which the prior context contains retrieval cues. Other accounts maintain that the N400 component is also a signature of *semantic integration or “unification” processes* (Hagoort et al., 2009). The integration view of the N400 holds that the amplitude of the N400 is modulated by the ease of integrating lexically retrieved information accessed from the current word into the prior context. The prior context can be constituted by a single word (Holcomb, 1993), a sentence (Kutas and Federmeier, 2000) or, with respect to our current aims, by a discourse (George et al., 1994; Van Berkum et al., 1999, 2003).

As for the latter, in some experiments a conflict was generated between discourse-level information and locally supplied semantic constraints. The results of these experiments were mixed though. Some studies reported that in the fictitious context of a cartoon-like story about an amorous peanut, the anomalous sentence “the peanut was in love” was processed more easily—i.e., it elicited a smaller N400—than the more conventional sentence “the peanut was salted” (Nieuwland and Van Berkum, 2006; see also Filik and Leuthold, 2008). However, other studies suggested that the context cannot override the briefly disruptive effects of local semantic violations. For example, in the fictional context of a Harry Potter story, a sentence such as “Harry used a book to teach the tough bread” is still more problematic than “Harry used a microwave to heat the tough bread” (see also Hald et al., 2007; Warren et al., 2008). The discrepancies between these studies may be taken to reflect differences in how strongly the context constrains the interpretation of the critical phrases (see the related discussion in Nieuwland, 2013). From the point of view of the Generative Lexicon (Pustejovsky, 1995), these two studies can be seen as addressing the formal component in the lexicon of nouns (*peanut* and *tough bread* are inanimate in the lexicon, but they can be interpreted as animate thanks to contextual effects). Focusing on the telic rather than the formal component in the lexicon of nouns, our study may contribute to this controversy, addressing the issue of whether discourse-level information can override the local semantic violation generated by combining a noun (e.g., *funnel*) with a telically incongruent verb (e.g., *hang*).

As for the debate concerning the level of embodiment of comprehension, to our knowledge only one study so far has used ERPs to investigate the role of affordances in sentence comprehension (Chwilla et al., 2007). This study has shown that some combinations that were consistent with objects' affordances such as “They let the canoe into the water and paddled with Frisbees” were easier to process and led to less negative N400 compared to combinations that violated affordances such as “They let the canoe into the water and paddled with pullovers.” Based on the resemblance, in terms of the waveform and the timing of the effect, between the N400 component enhanced by the violation of affordances and the standard N400 effect enhanced by violations of semantic expectations, the authors argued that integrating affordances with sentence meaning occurs with the same ease as integrating semantic knowledge. No one, however, has so far explored *ad-hoc* affordances in relation to telic components. Doing so, our study aims at contributing to both debates, the one concerning the role of contextual factors in determining sentences' truth conditions and the one related to the role of affordances in semantic processing. The predictions to be tested in the experiment are the following.

*Semantic minimalism*, leading to a two-step model, assumes that the conflict between lexically specified telic components and contextually provided *ad-hoc* affordances is resolved only after the meaning of a sentence is generated^3^. This assumption leads to predictions regarding the N400 component that can be phrased differently according to the preferred functional interpretation of the N400 component. Given that we measure the N400 on the verb succeeding the noun, the interpretation of the N400 as reflecting ease of lexical retrieval provides us with a measure of the ease with which the lexical value of the verb is retrieved from semantic memory. According to Semantic Minimalism, the unmodulated telic component (*pour*) of the noun (*funnel*) is always uploaded from the lexicon into working memory regardless of the discourse context. That is, the retrieval of the lexical value of the telically congruent verb (*pour*) will always be facilitated whereas the retrieval of the lexical value of the telically incongruent verb (*hang*) is not. As a consequence, the N400 component measured on the telically congruent verb should be lower than the one measured on the telically incongruent verb, regardless of the discourse context.

On the other hand, *Truth-conditional Pragmatics*, in line with a single-step model, assumes that the conflict between lexically specified telic components and contextually provided *ad-hoc* affordances is resolved already in the process of sentence meaning composition. Thus, when the sentence “She uses the funnel to hang her coat” is preceded by a discourse inducing the *ad-hoc* affordance of hanging for the funnel, this should directly modulate the semantic contribution the noun makes to sentence meaning. Thus, the telic component (*pour*) of the noun (*funnel*) will not be uploaded from the lexicon into working memory. The retrieval of the lexical value of the telically congruent verb will hence not be facilitated. Consequently, in the *ad-hoc* affordance-inducing context the N400 component measured on the telically congruent verb (*pour*) should not be necessarily lower than the one measured on the telically incongruent verb (*hang*). Moreover, the N400 component measured on the telically congruent verb (*pour*) should be more negative in the *ad-hoc* affordance-inducing context than in the neutral context.

Given the lexical retrieval view of the N400, the predictions of Semantic Minimalism and Truth-conditional Pragmatics for different comparisons will then be the following.

In the neutral context both Semantic Minimalism and Truth-conditional Pragmatics predict that the retrieval of the lexical value of the telically congruent verb will be facilitated, because the telic component (*pour*) of the noun (*funnel*) is uploaded from the lexicon into working memory. Thus, the N400 component measured on the telically congruent verb will be lower than the N400 component measured on the telically incongruent verb. However, in the *ad-hoc* affordance-inducing context the predictions of Semantic Minimalism and Truth-conditional Pragmatics differ with regard to the retrieval of the lexical value of the telically incongruent verb (*hang*). For, Truth-conditional Pragmatics assumes that the *ad-hoc* affordance of hanging is present in working memory and will directly affect the lexical retrieval for upcoming words. Consequently, the N400 component measured on the verb *hang* should be lower in the *ad-hoc* affordance-inducing context relative to the neutral context for Truth-conditional Pragmatics rather than for Semantic Minimalism.

Turning to the integration view of the N400, what is the semantic integration problem to be solved? To compose the meaning of the sentence, the meanings of the noun *funnel* and the verb *hang/pour* have to be integrated into the meaning of the complex phrase “uses the funnel to hang/pour….”

Semantic Minimalism and Truth-conditional Pragmatics lead to different predictions as to the difficulty of this integration. According to the integration view of the N400, the more difficult the integration of a certain semantic component is, the more negative the N400 component measured on the corresponding word should be.

According to Semantic Minimalism what the meaning of the word *funnel* contributes to the meaning of the complex phrase always includes the generic affordance stored as the telic component in the lexicon.

Since according to Semantic Minimalism the context does not modulate the meaning of the noun *funnel*, the integration of the telically congruent noun-verb combination (*funnel-pour*) into a complex phrase will always be easier than the integration of the telically incongruent noun-verb combination (*funnel-hang*). The reason is that Semantic Minimalism holds the claim of bottom-up compositionality, that is, that sentence meaning is composed from lexical values prior to any modulation by contextual factors. This especially means that the contextually introduced *ad-hoc* affordance has no modulatory effect. Accordingly, Semantic Minimalism predicts that (1) the N400 components measured on the telically congruent verb will not differ between the neutral and the *ad-hoc* affordance-inducing context; (2) the N400 components measured on the telically incongruent verb will not differ between the neutral and the *ad-hoc* affordance-inducing context; (3) the N400 component measured on the telically incongruent verb will be higher than that measured on the telically congruent verb in the neutral context; (4) the N400 component measured on the telically incongruent verb will be higher than that measured on the telically congruent verb in the *ad-hoc* affordance-inducing context.

In contrast, according to Truth-conditional Pragmatics what the meaning of the word *funnel* contributes to the meaning of the complex phrase need not contain the generic affordance represented as the telic component in the lexicon, if instead it includes an *ad-hoc* affordance introduced in context. As a consequence, the ease of integrating the meanings of the noun and the verb into the meaning of the complex phrase depends on whether and what *ad-hoc* affordance has been incorporated into the meaning of the noun. Whereas, in a neutral discourse context, the telically congruent noun-verb combination should be easier to integrate into the complex phrase than the telically incongruent noun-verb combination, in the *ad-hoc* affordance-inducing context the situation is reversed. Here, the telically incongruent noun-verb combination should be easier to integrate than the telically congruent noun-verb combination.

Thus, the following predictions are made by Truth-conditional Pragmatics: (1) in the neutral context, an enhanced N400 should be elicited for the telically incongruent verb compared to the telically congruent verb; (2) in the *ad-hoc* affordance-inducing context an enhanced N400 should be elicited for the telically congruent verb compared to the telically incongruent verb; (3) an enhanced N400 should be elicited for the telically incongruent verb in the neutral context compared to the *ad-hoc* affordance-inducing context; and (4) an enhanced N400 should be elicited for the telically congruent verb in the *ad-hoc* affordance-inducing context compared to the neutral context.

As we have already clarified above, the current experiment is not designed to adjudicate between a lexical retrieval view of the N400 or a semantic integration view. However, regardless of whether one assumes one interpretation or the other, a further question arises for Truth-conditional Pragmatics. Given that context modulates the meaning of nouns, what aspect of the context is exactly responsible for this modulation? We explore two possible answers to this question, which correspond to the different predictions made by the amodal-symbolic and embodied-simulative views of meaning. According to the amodal-symbolic account, the modulation is due merely to the symbolic meaning of the words and phrases in the context, whereas the embodied-simulative view maintains that the modulatory effect is due to the situation described by the context and as mentally simulated by the comprehending subject.

In order to investigate this issue, we used LSA and determined the semantic similarity values (SSVs) between the test sentence including the complex phrase (“uses the funnel to pour/hang…”) and the discourse context. We determined this value and kept it invariant across the experimental conditions. If the modulation effect predicted by Truth-conditional Pragmatics still occurs, it cannot be merely due to the symbolic meaning of the words and phrases in the linguist context but has to be explained on the basis of the situation described by the context and mentally simulated by the comprehending subject. This would count as indirect evidence against the amodal-symbolic view and in favor of the embodied-simulative view. Even though in some fields—e.g., psychology—the embodied-simulative account is already rather well-established, in other fields—e.g., philosophy, formal semantics and linguistics—the amodal-symbolic account is still predominant. Thus, providing some evidence against this view can help to advance the debate and increase our theoretical understanding of the subject matter. Furthermore, insofar as LSA, in line with the amodal-symbolic account, is used as a model to provide a measure of semantic similarity, evidence that SSVs cannot be a predictor of semantic expectancy and, hence, of the modulations of the N400, can be informative also for those primarily interested in the psychological repercussions of our findings.

## Materials and methods

### Participants

Twenty-two right-handed native speakers of Italian (13 males; mean age = 29, 2 years) participated in this study. All had normal or corrected-to-normal vision. None of the subjects had any neurological or psychiatric disorder, had experienced any neurological trauma, or used neuroleptics. They were paid for their participation.

### Stimuli

We created 80 noun-verb combinations. Half of them were obtained by combining nouns with telically congruent verbs (Telic combinations) while the other half was generated by replacing the telically congruent verbs with telically incongruent verbs (NonTelic combinations). We then created 80 test sentences using Telic and NonTelic combinations. The test sentences were preceded by a Neutral discourse context (N_CON) or by an *ad-hoc* affordance-inducing context (A_CON), for a total of 160 stories presented in four experimental conditions in a 2 × 2 design (see Table 1): (1) N_CON-Telic, (2) N_CON-NonTelic, (3) A_CON-Telic, (4) A_CON-NonTelic. As shown in Table 1, the noun occurs twice in each trial, however the use of the Italian determinative article preceding the second occurrence (e.g., *il, la*) prevents the reader from assuming that a new object has been introduced, indicating instead that the same object is mentioned again.

**Table 1:** **Example of experimental stimuli with English translation**.

	**TELIC**	**NONTELIC**
N_CON	Chiara si è attrezzata con un imbuto per fare in casa un piccolo esperimento di chimica e, a tal fine, ha messo un colorante nell'acqua.	Chiara si è attrezzata con un imbuto per fare in casa un piccolo esperimento di chimica e, a tal fine, ha messo un colorante nell'acqua.
	Una volta fatto ciò,	Essendo un tipo originale,
	usa l'*imbuto* per **versare** l'acqua in un contenitore.	usa l'*imbuto* per **appendere** il cappotto.
	Clare got herself a funnel to perform a little chemistry experiment at home and to this end she put some dye in water. Once she has done so, she uses the funnel to pour water into a container.	Clare got herself a funnel to perform a little chemistry experiment at home and to this end she put some dye in water. Being an unconventional person, she uses the funnel to hang her coat.
A_CON	Chiara ha un imbuto in più e, dopo aver deciso cosa farne, lo inchioda per bene al muro lasciando la parte più stretta rivolta verso l'esterno.	Chiara ha un imbuto in più e, dopo aver deciso cosa farne, lo inchioda per bene al muro lasciando la parte più stretta rivolta verso l'esterno.
	Una volta fatto ciò,	Essendo un tipo originale,
	usa l'*imbuto* per **versare** l'acqua in un contenitore.	usa l'*imbuto* per **appendere** il cappotto.
	Clare has an extra funnel and, after having decided what to do with it, she glues it to the wall leaving the narrow end facing outward. Once she has done so, she uses the funnel to pour water into a container.	Clare has an extra funnel and, after having decided what to do with it, she glues it to the wall leaving the narrow end facing outward. Being an unconventional person, she uses the funnel to hang her coat.

*The table illustrates a 2 × 2 design, in which two categories of noun-verb combinations, Telic and NonTelic, are combined with two categories of discourse context, Neutral (N_CON) and ad-hoc affordance-inducing context (A_CON). In the table, the discourse context is shown in black, the internal context preceding the target clause is in blue and the target clause is in red. The test sentence is composed of the internal context and the target clause. The cue word is in bold while the noun preceding the cue word is in italics*.

The ERPs recording was time-locked to the onset of the cue words, which were always verbs. The cue verbs always occurred in the midst of the sentence to control for “wrap-up effects” at sentence-final words reported in several ERPs studies (e.g., Hagoort, 2003). The cue verbs in the Telic and NonTelic combinations were setwise matched on the following features: word length, number of syllables, mean word frequency (see Table 2). The Neutral contexts and the *ad-hoc* affordance-inducing contexts were pair-wise matched for number of words.

**Table 2:** **Features of the cue words in Telic and NonTelic combinations**.

	**Number of letters (mean)**	**Number of syllables (mean)**	**Mean frequency^a^**
Telic combination	7.575	2.95	11,732.73
NonTelic combination	7.575	3	11,822.68

a *CORPUS “La Repubblica” ca. 331 milions tokens*.

### Latent semantic analysis

We translated the experimental stimuli into English and applied to them three separate Latent Semantic Analyses (Tables 3–**5**) using the online resources available at http://lsa.colorado.edu/. First, we controlled that the SSVs for Telic combinations (e.g., funnel-pour) were indeed higher than those for NonTelic combinations (e.g., funnel-hang). Specifically, we carried out a “Pairwise [term to term] comparison” using the tasaALL space, which corresponds to the first year college level. For this space, the LSA matrix is based on the occurrence of 92,409 unique terms in 37,651 contexts selected from texts, novels, newspaper articles, and other information. As Table 3 shows, the difference in SSVs between the two sets of noun-verb combinations was significant *t*_(39)_ = 5.449, *P* < 0.001, CI = 0.16 ± 0.06, indicating that in Telic combinations the noun and the verb are more semantically related than in NonTelic combinations.

**Table 3:** **Semantic Similarity Values (SSVs) between the noun and the verb in Telic and NonTelic combinations**.

**Telic combinations (e.g., funnel—pour)**	**NonTelic COMBINATIONS (e.g., funnel—hang)**	
0.30	0.13	*t*_(39)_ = 5,449, *p* < 0.001

*The table shows that the SSVs between the noun and the telically congruent verb (Telic combinations) are significantly higher than those between the noun and the telically incongruent verb (NonTelic combinations)*.

A possible problem with using this method to compute semantic relatedness is that LSA is based on American English. Thus, we had to translate the experimental stimuli from Italian to American English. The issue is whether subtle but important differences between the translated stimuli and the original Italian stimuli might affect LSA results. Even though we cannot completely rule out this possibility, we complemented the LSA analysis with an additional EEG experiment, reported in Appendix A (see Supplementary Figure 1). The aim of this EEG experiment was two-fold. First, we wanted to test the prediction of amodal-symbolic theories which, on the basis on the LSA result, expect a difference in the semantic expectancy for the telically congruent verb compared to the telically incongruent verb. The N400 component should be enhanced for the latter but not for the former. Second, we wanted to test whether the results of the LSA analysis, based on translations of the original stimuli, was consistent with the result of the EEG experiment, in which the original Italian stimuli were used. We reasoned that if they happened to be consistent, this would support the validity of the LSA analysis, despite the possible limitations given by the translation procedure.

However, in the present experiment, Telic and NonTelic combinations were not presented in isolation. They were embedded within a test sentence which was, in turn, preceded by a discourse context. We carried out then two other analyses using LSA to ascertain that apart from the difference in the SSVs between the noun and the verb, there were no other differences in the SSVs among the four experimental conditions. First, we run a “Pairwise [document-to document] comparison” using the tasaALL space to ascertain that the SSVs between the test sentence and each type of discourse context did not differ among conditions. Second, given that the test sentence was composed of a target clause introduced by a short internal context (mainly, adverbial phrases), we also carried out a “Pairwise [term-to document] comparison” using the tasaALL space to control that the SSVs between the cue word and the sets of words in the preceding internal context did not differ among the experimental conditions.

As illustrated by Table 4, the mean SSVs between the test sentence and the context were almost the same across all four experimental conditions. In particular, there was no significant difference in the SSVs obtained comparing the discourse context and (1) the test sentences presenting Telic combinations, *t*_(39)_ = 0.496, *p* > 0.05, and (2) the test sentences presenting NonTelic combinations, *t*_(39)_ = 0.288, *p* > 0.05. This indicates that the sets of words in the Telic and NonTelic test sentences were, respectively, matched in terms of semantic relatedness to the sets of words in the Neutral and *ad-hoc* affordance-inducing contexts.

**Table 4:** **Semantic Similarity Values (SSVs) for the test sentence and the discourse context**.

**Test sentence**	**SSVs for test sentences compared to discourse contexts**		
	**N_CON-**	**A_CON-**	
Telic	0.36	0.35	t(39) = 0.496, p > 0.05
NonTelic	0.34	0.34	*t*_(39)_ = 0.288, *p* > 0.05

*As shown by the table, there is no significant difference in the SSVs between the test sentence (using, respectively, Telic and NonTelic combinations) and the context (Neutral vs. ad-hoc affordance-inducing context)*.

Furthermore, as illustrated by Table 5, even though the internal context was changed across conditions just to improve readability, there was no effect due to that. Indeed, there was no significant difference in the SSVs between the cue word and the internal context in the Telic and NonTelic combinations, *t*_(39)_ = 1,550, *p* > 0.05, showing that the cue words were matched in terms of semantic relatedness to the sets of words in the internal contexts.

**Table 5:** **Semantic Similarity Values (SSVs) between the cue words and the internal contexts**.

	**Internal context**	**SSVs between cue words and internal contexts**	
Cue word	Telic	0.26	*t*_(39)_ = 1,550, *p* > 0.05
	NonTelic	0.20	

*The table shows that there is no significant difference between the SSVs obtained comparing the cue words with the internal contexts in the Telic and NonTelic conditions*.

### Procedure

Participants were seated in a sound-proof and electrically shielded cabin. After signing the informed consent and completing the Edinburgh handedness test, they were informed that they would be reading short stories, and were instructed to read for comprehension and minimize movements. No additional task demands were imposed. Word stimuli were presented in black font on a white background at the center of a computer monitor at a viewing distance of 80 cm.

Each trial started with a fixation cross (1,300 ms) followed by the presentation of the discourse context shown all at once. Context duration in ms was computed as (*n* × 400 ms), n = number of phrases, with a five phrases maximum, to mimic natural reading times while avoiding, at the same time, any effect due to individual differences in reading time. After a fixation cross of 1,300 ms, the phrase by phrase presentation of the internal context started, with a 450 ms phrase duration and a variable random 250–450 ms inter-stimuli interval. The target clauses followed word-by-word. Each word was displayed at the center of the screen for 450 ms, with a 250–450 ms pseudo-random blank interval between successive word presentations. The pseudo-randomization of the inter-stimuli interval was chosen to ensure that the cue word was always followed by a 450 ms interval (which was necessary to record 1,200 ms epochs), while avoiding an excessively slow presentation of the target sentences if this interval would have been held constant after the presentation of each word or any effect on the processing of the cue words if the interval following them would have been longer than the interval following the presentation of the non-cue words. Sentence final words were followed by a full stop, as shown in Table 1. They were followed by a blank interval of 200 ms after which the next trial could start. After the presentation of successive blocks of 30 sentences, a simple yes/no question was presented for 3 s (e.g., “Is the story plausible?” or “Can you imagine the scenario?”). These questions were presented to keep participants more alert during the experiment and were randomly distributed across the trials (including the filler stories: see below) and the participants.

Two trial lists were used, such that the cue word appeared only once in a given list. For the first list, 40 test sentences with Telic combinations and 40 test sentences with NonTelic combinations were presented, introduced by either a Neutral or an *ad-hoc* affordance-inducing context (20 N_CON-Telic, 20 A_CON-Telic, 20 N_CON-NonTelic, and 20 A_CON-NonTelic), and were randomly mixed with 40 filler stories. The second list was derived from the first by replacing all the test sentences containing Telic combinations with their NonTelic counterparts and vice versa. The 40 filler stories described scenarios about some character engaged in some activity, so they were similar to the experimental scenarios except that contained referentially ambiguous noun phrases or errors in subject-verb agreement. The total of 120 stories was divided into four blocks separated by a break, the duration of which was determined by the participant. Total time-on-task was ~40 min. Subjects completed the task across two separated sessions. In the second session they were assigned to the list that they did not see in the first session, such that at the end of the two sessions each subject had read 160 stories (40 per condition) excluding fillers. We required each participant to undertake the second session only after a minimum interval of 2 weeks from the first one. The data from the two sessions were then pulled together in the Brain Vision Analyzer software before processing them. Such a relatively long interval should be sufficient to avoid any effect of word repetition and to minimize other potential memory effects between the two sessions, however the procedure used does not allow us to quantitatively determine the potential change in the ERP effects across the two testing sessions.

### EEG recording and data processing

A BrainAmp™ acticap system was used to record the electroencephalogram (EEG) from 66 active electrodes including four electro-oculogram (EOG) electrodes for monitoring horizontal and vertical eye movements. EEG and EOG signals were digitized at 1000 Hz and with an online band-pass filter of 0.53–70 Hz. Impedance was kept below 5 kΩ for scalp electrodes and below 10 kΩ for EOG electrodes. The EEG data were processed using Brain Vision Analyzer 2.0 software. All EEG channels were re-referenced off-line to the average of the left and right mastoid channels (TP9 and TP10) and filtered with a high cutoff of 30 Hz, 12 dB/oct. An automatic raw data inspection rejected trials with amplitude differences exceeding 200 μV in a 200 ms time interval and with activity lower than 50 μV in a 100 ms interval. Four channels (i.e., Fp1, Fp2, AF7, AF8) were disabled due to excessive artifacts. Ocular artifacts were corrected by means of a procedure based on independent component analysis (ICA). Single-trial waveforms were separately extracted during 1,200 ms epochs (starting 200 ms before critical word onset), averaged, baseline corrected to 200 ms pre-stimulus onset and screened for artifacts. Segments with potentials exceeding ±90 μV were rejected. One participant was excluded due to excessive artifacts (trial loss = 50%). For the remaining 21 participants, average ERPs were computed over artifact-free trials per condition (average percentage of included trials = 96%, range = 72–100% across the four conditions).

### Statistical analysis

A preliminary 2(Context: N_CON, A_CON) × 2(Combination: Telic, NonTelic) repeated measures analysis of variance (ANOVA) was performed in consecutive 100 ms time windows between 200 and 600 ms after critical word onset. The selected epoch encompasses the standard time interval during which N400 experimental effects were found to be most pronounced (e.g., Kutas et al., 2006) and allowed us to examine possible effects of the experimental conditions on other processes preceding or following those eliciting the N400. All EEG electrode sites were included in this analysis, calculating average changes in amplitude over all channels per condition.

To investigate potential topographic changes in the distribution of the N400 effects, we adopted a systematic columnar “pattern of analyses” similar to that used in other studies (e.g., Ditman et al., 2008; Paczynski and Kuperberg, 2012). This approach allows to detect differences in the distribution of effects along the anterior-posterior (AP) axis of the scalp, and differences across the two hemispheres at lateral electrode columns. ERP amplitudes measured at midline electrodes were subjected to an ANOVA with the variables Context (two levels: N_CON, A_CON), Combination: Telic, NonTelic) and Anterior-Posterior (AP) distribution (six levels: Fz, FCz, Cz, CPz, Pz, POz). For the analysis of the peripheral columns, electrodes were divided along left–right, medial–lateral, and dorsal–ventral dimensions. This resulted in eight columns. The four columns of the left hemisphere were defined as follows: dorsal-medial (F1, FC1, C1, CP1, P1), dorsal-lateral (AF3, F3, FC3, C3, CP3, P3, PO3), ventral-medial (F5, FC5, C5, CP5, P5), ventral-lateral (F7, FT7, T7, TP7, P7). Four analogous columns were defined for homolog electrodes located over the right hemisphere. The ANOVAs for peripheral sites had as variables Context (two levels: N_CON, A_CON), Combination (two levels: Telic, NonTelic), Hemisphere (two levels: left, right), Anterior-Posterior (AP) distribution (with the number of levels depending on the number of electrode sites in each column).

A follow-up ANOVA was performed when interactions with the AP factor were found. This analysis involved specifically a predetermined region over centro-parietal sites at which the N400 is maximal (e.g., Kutas et al., 2006). In this case, a 2(Context: N_CON, A_CON) × 2(Combination: Telic, NonTelic) × 7 (Electrodes: CP1, CP2, CPz, Pz, P1, P2, POz) ANOVA was conducted. Bonferroni-adjusted planned comparisons were performed to decompose the effect of trial type in this region. The Greenhouse-Geisser correction was applied to F tests with more than one degree of freedom in the numerator to protect against Type 1 errors resulting from violations of sphericity (corrected *p*-values and degrees of freedom are reported).

## Results

**Figure 1:**
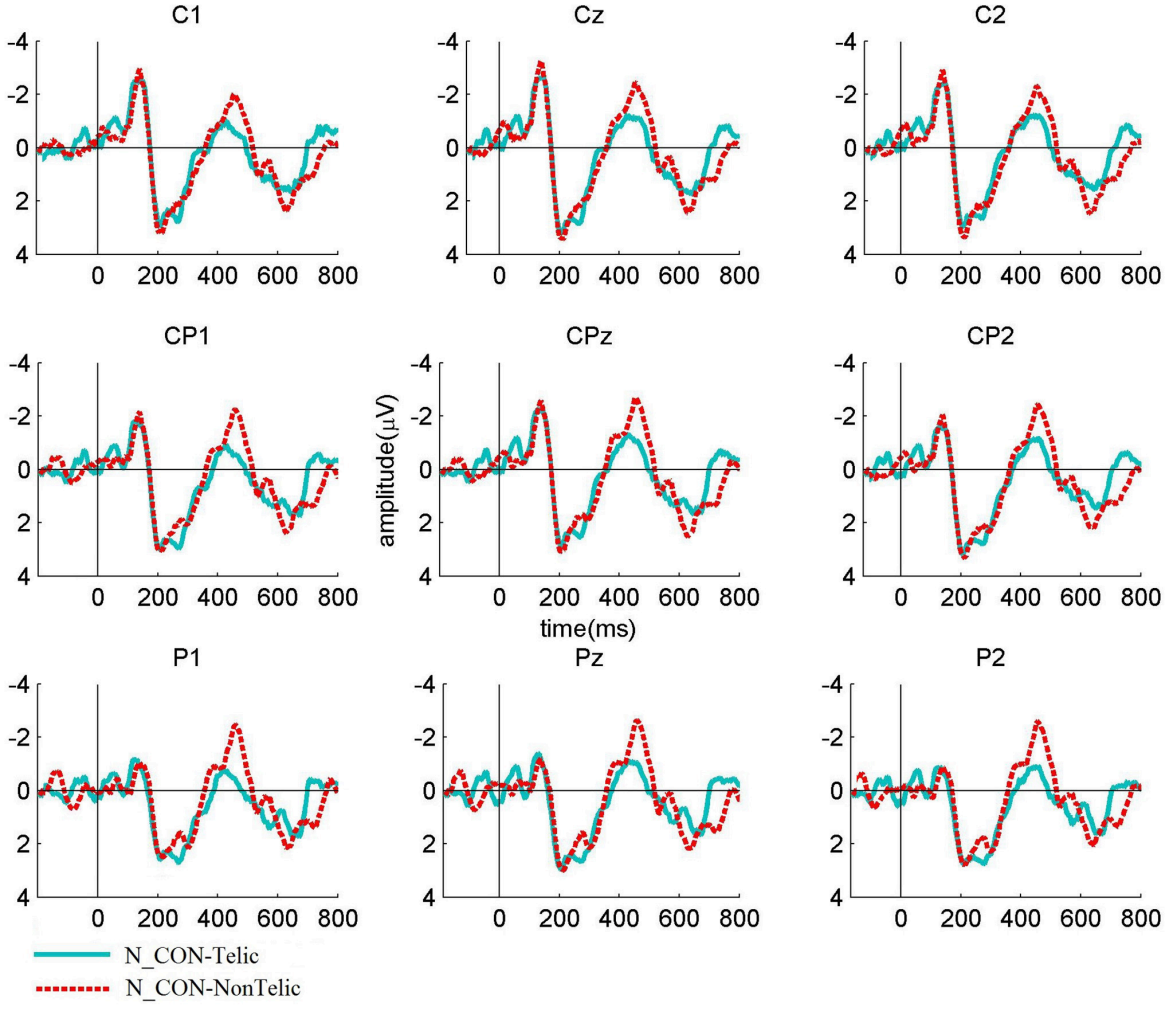
**Grand average waveforms elicited by the verbs in Telic vs**. NonTelic combinations in Neutral contexts (N_CON) for nine centro-parietal channels. The waveforms show a significant N400 effect.

**Figure 2:**
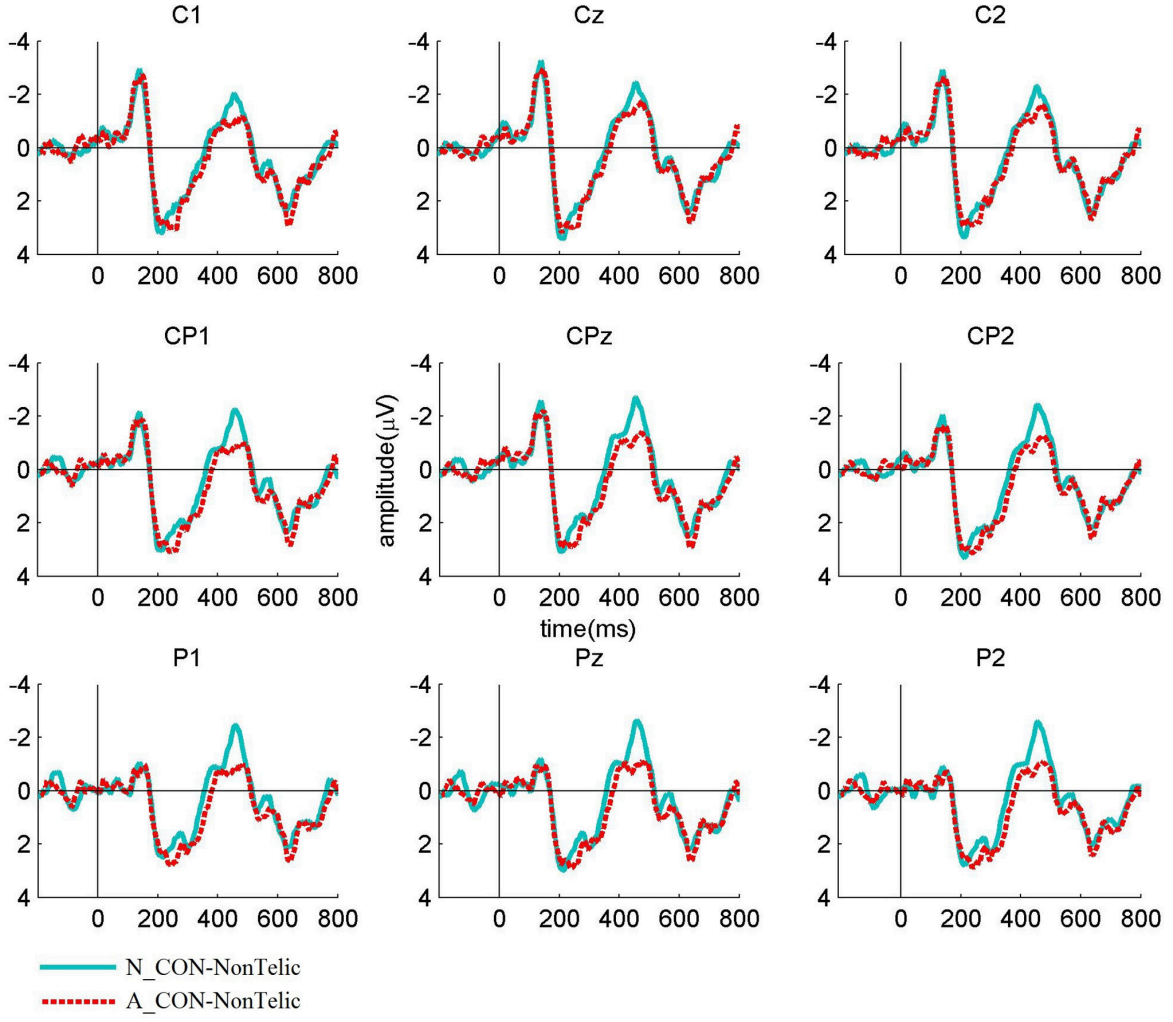
**Grand average waveforms elicited by the verbs in NonTelic combinations for Neutral contexts (N_CON) vs. ***ad-hoc*** affordance-inducing contexts (A_CON) over nine centro-parietal channels**. The waveforms show that the N400 component for the verbs in NonTelic combinations is significantly reduced in A_CON compared to N_CON.

**Figure 3:**
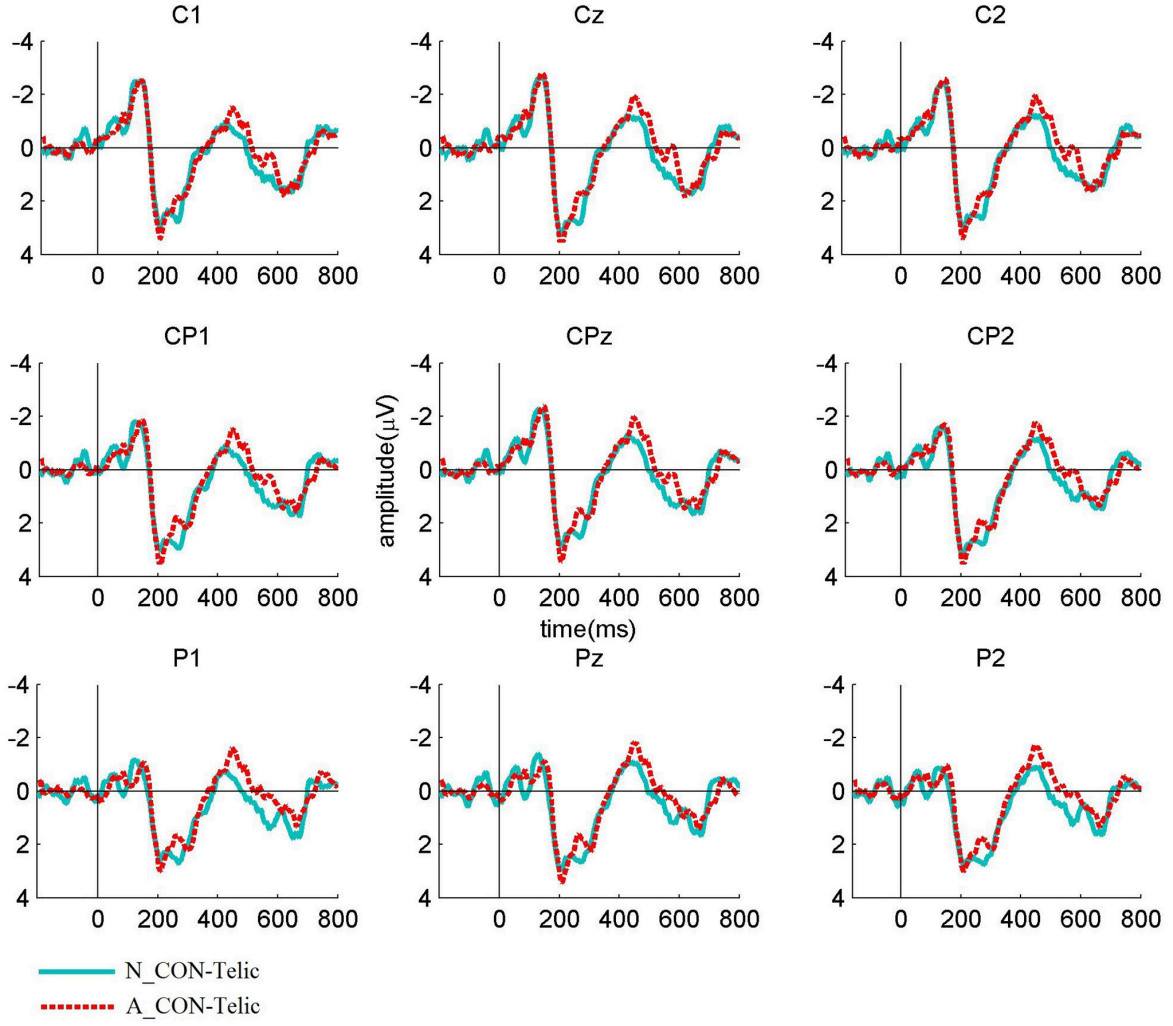
**Grand average waveforms elicited by the verbs in Telic combinations for Neutral contexts vs. ***ad-hoc*** affordance-inducing contexts over nine centro-parietal channels**. The waveforms show that the N400 component for the verbs in the Telic combinations is significantly enhanced in A_CON compared to N_CON.

**Figure 4:**
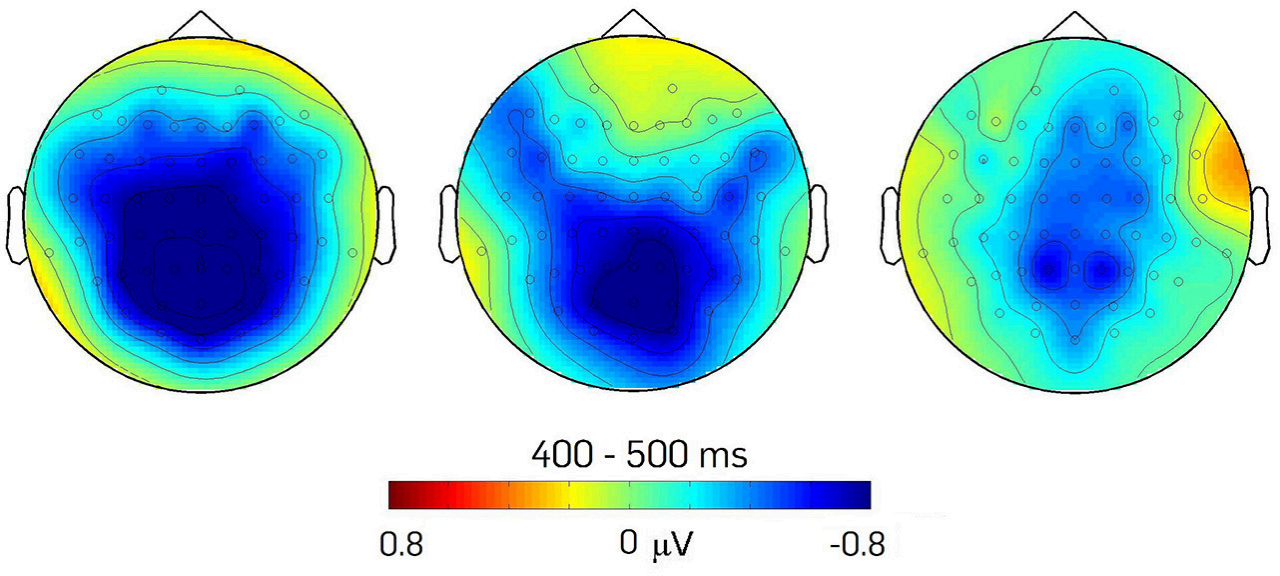
**Scalp distribution of the effects of modulation of the N400 component**. The topographical maps show: (a) the difference between the verbs in Telic and NonTelic combinations in Neutral contexts (N_CON-NonTelic *minus* N_CON-Telic); (b) the difference between the verbs in NonTelic combinations in Neutral vs. *ad-hoc* affordance-inducing context (N_CON-NonTelic *minus* A_CON-NonTelic); (c) the difference between the verbs in Telic combinations in Neutral vs. *ad-hoc* affordance-inducing context (A_CON-Telic *minus* N_CON-Telic).

The Figures 1–**3** show the grand average ERP waveforms elicited by telically congruent and telically incongruent verbs in the conditions of interest. For readability reasons, the figures show only 800 ms of the recorded 1,200 ms epochs. More precisely, Figure 1 shows the grand average waveforms elicited by telically congruent and telically incongruent verbs in N_CON. As can be seen from the figure, in N_CON a more negative N400 component is elicited by a telically incongruent verb than a telically congruent verb. Figure 2 depicts the grand average waveforms elicited by telically incongruent verbs in N_CON and A_CON. The figure shows that the N400 component elicited by a telically incongruent verb is reduced in N_CON compared to A_CON. Finally, Figure 3 illustrates the grand average waveforms elicited by telically congruent verbs in N_CON and A_CON, and shows an enhanced N400 for a telically congruent verb in A_CON compared to N_CON. The scalp distribution of the effects is shown in Figure 4.

The omnibus ANOVA revealed that the interaction between Context and Combination was significant in the 400–500 ms time interval, *F*_(1, 20)_ = 5.110, *p* < 0.05. No other significant effects or interactions were found in the other time windows [200–300 ms: *Fs*_(1, 20)_ < 0.186, *ps* > 0.05; 300–400 ms: *Fs*_(1, 20)_ < 1.044, *ps* > 0.05; 500–600 ms: *Fs*_(1, 20)_ < 2,974, *ps* > 0.05]. Following-up on this effect, we systematically explored its topography. We established that the interaction between Context and Combination was still significant in the Midline analysis, *F*_(1, 20)_ = 5.202, *p* < 0.05. We also found a significant interaction between Context and AP distribution, *F*_(1.936, 38.712)_ = 3.724, *p* < 0.05 and a three-way Context × Combination × AP distribution interaction, *F*_(1.862, 37.249)_ = 4.305, *p* < 0.05.

Significant interactions between Context and Combination were found in dorsal-medial, *F*_(1, 20)_ = 4.395, *p* < 0.05 and dorsal-lateral columns as well, *F*_(1, 20)_ = 4.756, *p* < 0.05. Along the dorsal-medial sites there was also a significant three-way interaction between Context, Combination and AP distribution, *F*_(1.466, 29.317)_ = 8.762, *p* < 0.005, whereas at the dorsal-lateral sites the Context × AP distribution interaction was only trend-wise significant, *F*_(1.726, 34.520)_ = 2.938, *p* = 0.073. No interactions with Hemisphere were found, indicating that the effect was equally distributed between the two hemispheres. No other main effects or interactions were found at the ventral-medial sites, where the interaction between Context and Combination only approached significance, *F*_(1, 20)_ = 3.866, *p* = 0.063, nor at the ventral-lateral sites. The interactions with AP distribution indicated a larger effect over centro-posterior electrodes in the Midline analysis and over dorsal-posterior sites in the peripheral columns.

A follow-up ANOVA of the predetermined N400 region showed a significant Context × Combination interaction, *F*_(1, 20)_ = 11.267, *p* < 0.005. There was no interaction with electrodes in this region, possibly indicating that the effect was distributed across all sites.

**Figure 5:**
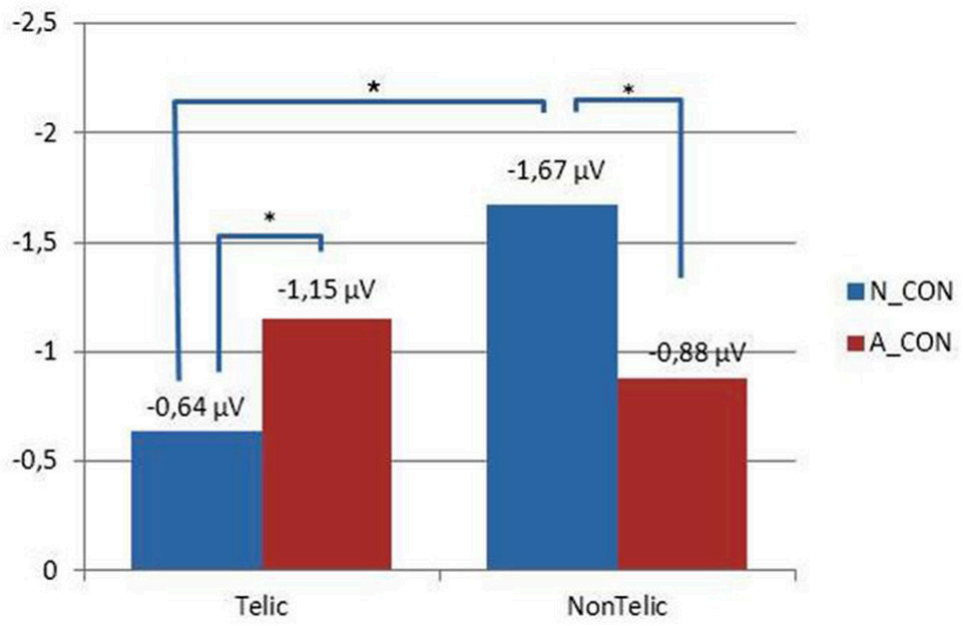
**Crossing over regarding the N400 component**. Relative to the neutral context, the *ad-hoc* affordance-inducing context significantly enhances the N400 component for Telic noun-verb combinations whereas it significantly reduces the N400 component for NonTelic noun-verb combinations.

Figure 5 shows the mean amplitudes of the N400 component for each condition of interest. As illustrated by the graph, we observed a crossing-over regarding the direction of the N400 effects. Relative to the Neutral context, the *ad-hoc* affordance-inducing context enhances the N400 component for a telically congruent verb whereas it reduces the N400 component for a telically incongruent verb^4^. Planned comparisons allowed decomposing the effect according to trial type.

First, replicating the standard N400 effect to semantic violations (Kutas and Hillyard, 1984), we found that in N_CON, a telically incongruent verb [M(N_CON-NonTelic) = −1.67 μV, *SD* = 1.69 μV] elicits a more negative N400 than a telically congruent verb [M(N_CON-Telic) = −0.64 μV, *SD* = 1.46 μV, *t*_(20)_ = 3.069, *p* = 0.006, CI = 1.03 ± 0.70]. Second, the N400 component for a telically incongruent verb is significantly more negative in N_CON than A_CON [M(A_CON-NonTelic) = −0.88 μV, *SD* = 1.64 μV, *t*_(20)_ = −2.745, *p* = 0.012, CI = −0.79 ± 0.60]. Third, a more negative N400 component was elicited by a telically congruent verb in A_CON (M (A_CON-Telic) = −1.15 μV, *SD* = 1.13 μV) than in N_CON, *t*_(20)_ = 2.276, *p* = 0.034, CI = 0.51 ± 0.47. Finally, in A_CON the mean of N400 component measured on the telically congruent verb was more negative than that measured on the telically incongruent verb. However, this difference was not significant, *t*_(20)_ = 0.964, *p* = 0.34, possibly because of the combined and contrasting effect of semantic similarity and motor simulation.

## Discussion

The aim of this study was to investigate the time-course of the interaction between the lexically specified telic component of a noun (i.e., the function or the purpose of the object to which the noun refers) and an *ad-hoc* affordance in the situation introduced by the preceding linguistic discourse. By investigating this issue, we aimed at exploring a two-dimensional theory space in which theories of linguistic comprehension can be positioned according to (i) the way and the time-course in which contextual factors influence the intuitive truth-conditions of sentences and (ii) whether and to what extent comprehension involves sensory, motor and emotional processes. For simplicity, we focused on the poles of each dimension even though the actual theory space is much more complex than we represented. We used the N400 effect as an ERP paradigm to test the predictions of the different theories with respect to the dimensions of interest.

Regarding the first dimension, in the philosophy of language it has become a hotly debated issue between Semantic Minimalism (Borg, 2004, 2012) and Truth-conditional Pragmatics (Recanati, 2012), as well as intermediate positions (e.g., Stanley, 2007), whether pragmatic aspects of the discourse directly interact with meaning components retrieved from the lexicon as well as with any further node in the sentence meaning composition tree (for a related discussion see also Spychalska et al., 2016).

What is at stake here is how to interpret the notion of compositionality, according to which the meaning of a complex expression is determined by the meanings of its syntactic parts and the way the parts are combined (Werning, 2004, 2005, 2012; Werning et al., 2012). Semantic Minimalism endorses a rigorous notion of bottom-up compositionality, in which the truth-evaluable semantic content of a sentence results from a rule-based combination of lexical-semantic features of the words in a sentence without taking into account contextual effects (Borg, 2004, 2012; Cappelen and Lepore, 2005). Before context information comes into play, according to the minimalist, the process of semantic composition has already determined the semantic content of the sentence. The role of the context at this level is limited to cases of indexicality and anaphor resolution and should be traceable to specific syntactic parts of the sentence (e.g., “now,” “this,” “he,” etc.). All other pragmatic processes involved in the interpretation of the utterance are secondary and presuppose an already determined semantic content. Linguistic interpretation is thus construed as a two-step procedure: only after a context-insensitive level of semantic content is completely generated, the content can be manipulated to accommodate the contextually construed interpretation of the utterance.

Truth-conditional Pragmatics challenges the rigorous notion of compositionality claiming that pragmatic enrichment is “free,” that is, any however remote information can in principle modulate the meaning of a linguistic expression at any stage in semantic composition. Accordingly, a situation introduced in a discourse preceding the sentence may result in the modulation of the meanings of words or phrases in the sentence and of the sentence itself (Cosentino et al., 2013). These modulations will then influence the intuitive truth-conditions of the sentence. This view, as developed for example by Recanati (2012) amounts to a weakening of the rigorous notion of compositionality by introducing context-dependent semantic flexibility by means of modulation. Evidence of top-down effects would then question the strong notion of bottom-up compositionality endorsed by Semantic Minimalism and speak in favor of a less rigorous notion of compositionality as assumed by Truth-conditional Pragmatics.

The results of our experiment speak to this debate showing that the amplitude of the N400 component is modulated by pragmatic aspects of the discourse context. Given that the N400 is commonly taken to reflect automatic processes of either lexical retrieval or semantic integration, these differences in its amplitude suggest that even remote information, namely an *ad-hoc* affordance of a situation discursively introduced, does indeed modulate the meaning of a word or phrase in a sentence before sentence meaning composition is completed (e.g., Wilson and Carston, 2007; see also, Asher, 2011). This finding is consistent with the results from other studies, which show similar contextual effects focusing on different linguistic phenomena. For instance, Bambini et al. (2016) have shown that the preceding context has the effect of reducing the amplitude of the N400 typically elicited by metaphorical expressions.

The direct contextual modulation of a noun's contribution to sentence meaning can be explained by two different mechanisms, which are consistent with two different functional interpretations of the N400. On one interpretation, the contextually provided *ad-hoc* affordances impede the retrieval of the noun's telic component from the lexicon into working memory. As a consequence, the retrieval of the lexical value of the telically congruent verb will not be facilitated, leading to the described effects on the amplitude of the N400. According to this hypothesis, changes in the amplitude of the N400 might also be expected due to individual differences in subjects' working memory ability. In particular, subjects with lower working memory capacity should have more difficulties in storing the contextually provided *ad-hoc* affordances in working memory, thus they should be less sensitive to contextual effects.

On the second interpretation, the *ad-hoc* affordance introduced in the context is directly incorporated into the meaning of the noun, thus contributing to the meaning of the complex phrase in which they are embedded. Consequently, telically incongruent noun-verb combinations are easier to integrate into the complex phrase than telically congruent noun-verb combinations. The current study was not designed to adjudicate between these two mechanisms. However, regardless of the specific mechanism through which contextual modulation is realized, our results show that such a modulation does indeed occur.

Whereas Semantic Minimalism is incompatible with most of these results, Truth-conditional Pragmatics clearly predicts those effects. The first result, that is, that in a neutral context a telically incongruent verb elicits a more negative N400 than a telically congruent verb, is actually predicted by both Semantic Minimalism and Truth-conditional Pragmatics in either functional interpretation of the N400. The other results can instead adjudicate between the two theories. In particular, the N400 component for a telically incongruent verb was found to be significantly more negative in a neutral context than in an *ad-hoc* affordance-inducing context. Assuming the functional interpretation of the N400 as reflecting the ease of lexical retrieval this result confirms the prediction of Truth-conditional Pragmatics, but disconfirms the prediction of Semantic Minimalism. For, Truth-conditional Pragmatics predicts that the N400 component measured on a telically incongruent verb should be more negative in the neutral than in the *ad-hoc* affordance-inducing context, whereas Semantic Minimalism predicts that the N400 should not differ between the two contexts. Given the interpretation of the N400 as reflecting the ease of semantic integration, the result disconfirms Semantic Minimalism, which predicts that the N400 component measured on a telically incongruent verb should not be different between the *ad-hoc* affordance-inducing context and the neutral context, whereas Truth-conditional Pragmatics allows for a difference. Finally, the finding that a more negative N400 component was elicited by a telically congruent verb in the *ad-hoc* affordance-inducing context than in the neutral context is predicted by Truth-conditional Pragmatics in either interpretation of the N400, whereas no difference is predicted by Semantic Minimalism.

On both interpretations of the N400, Semantic Minimalism predicts that in the *ad-hoc* affordance-inducing context the N400 measured on the telically incongruent verb should be more negative than that measured on the telically congruent verb. Given the interpretation of the N400 as indicating ease of semantic integration, Truth-conditional Pragmatics indeed predicts that in the *ad-hoc* affordance-inducing context the N400 measured on the telically congruent verb should be more negative than that measured on the telically incongruent verb, whereas Semantic Minimalism makes the reversed prediction, namely that in the *ad-hoc* affordance-inducing context the N400 measured on the telically incongruent verb should be more negative than that measured on the telically congruent verb. Given the interpretation of the N400 as indicating ease of lexical retrieval, Truth-conditional Pragmatics predicts that in the *ad-hoc* affordance-inducing context the N400 measured on the telically incongruent verb should not be more negative than that measured on the telically congruent verb. Semantic Minimalism, in contrast, predicts that the N400 component for the telically incongruent verb should be more negative than that for the telically congruent verb.

In sum, most of the predictions of Truth-conditional Pragmatics, in either interpretation of the N400, were confirmed by the experiment, and none of its predictions was disconfirmed. Looking just at the predictions of Semantic Minimalism which differed from the predictions of Truth-conditional Pragmatics, most of them have been disconfirmed by the experiment, and none has been confirmed. It should be noted that whereas our predictions focus on the N400 modulations occurring when the cue verb is processed in combination with the preceding noun, variations in the amplitude of the N400 component due to contextual influence, might occur already at the processing of the noun itself. Given our experimental setting, we cannot establish whether this is the case or not, so this potential limitation of the current study should be taken into account in future research.

Once established that contextual factors modulate the meaning of a word or a phrase in a sentence before sentence meaning composition is completed, it remains to be clarified what aspect of the context is exactly responsible for this modulation. We need to spell out how these results can contribute to the debate concerning our second dimension of interest: whether and to what extent comprehension involves sensory, motor, and emotional processes. More precisely, we need to clarify whether the contextual effect is merely due the symbolic meaning of the words and phrases in the context or to the situation described by the context and as mentally simulated by the comprehending subject.

In order to address this issue, we used Latent Semantic Analysis, which provides the amodal-symbolic account of meaning with a method of quantitatively determining meaning similarity and semantic relatedness in terms of Semantic Similarity Values (SSVs). On the basis of SSVs, LSA allows amodal-symbolic theories to predict the semantic expectancy of words in their linguistic contexts. Given that LSA is based on American English, we had to translate the experimental stimuli from Italian to American English. It is an open issue whether this might have affected the LSA results. However, we complemented the LSA analysis with a further EEG experiment reported in Appendix A, in which the original Italian stimuli were used, and found that the results of this experiment were consistent with the results of the LSA analysis. Thus, even though we cannot rule out the possibility that the translation might have affected the LSA results, the results of the complementary EEG experiment suggest that the mapping between the original stimuli and the translated ones was at least sufficient.

We measured the SSVs between a test sentence (which includes a complex phrase, e.g., “uses the funnel to pour/hang…”) and its preceding discourse context and made sure that there was no significant difference among the four experimental conditions. Thus, as far as LSA provides a proper measure of semantic similarity, the SSVs cannot be a predictor, in our experiment, of semantic expectancy and, hence, of the modulations of the N400. Given that the N400 modulations cannot be accounted for by appealing to differences in SSVs, one possibility is that the aspect of the context that is responsible for the modulation of the meaning of nouns is rather the motor information brought about by the *ad-hoc* affordances during the mental simulation of the situation described by the context. Even though this is an indirect way of testing the contrast between amodal-symbolic and embodied-simulative theories, the modulations of the N400 that we have observed cannot be accounted for by appealing to differences in the semantic similarity between the words in the context and those in the test sentence. One possibility is that the N400 modulations are consistent instead with a central tenet of most embodied-simulative accounts—either hybrid or full-blooded—according to which comprehension necessarily involves simulating the situation linguistically described in the context.

## Conclusion

The results of this ERP study challenge Semantic Minimalism, which holds that sentence meaning is composed from unmodulated lexical values prior to any influence by contextual factors. The reported N400 effects suggest that contextual factors do indeed modulate the meaning of a word or a phrase in a sentence before sentence meaning composition is completed. Thus, the results of this study are in line with Truth-conditional Pragmatics, which introduces context-dependent semantic flexibility by means of modulation. A further question addressed in our experiment is what aspect of the context is responsible for this modulation. Using Latent Semantic Analysis as a tool to quantitatively determine meaning similarity, we argued that the reported N400 effects cannot be explained in terms of differences in the semantic similarity between the words and phrases in different experimental conditions. The contextual modulation of the meaning of a word or a phrase in a sentence may rather be due to the motor information activated in the mental simulation of the situation described in the linguistic context. Thus, although indirectly, the results of this experiment challenge, in the case of the linguistic processing of affordances, the amodal-symbolic view and are consistent instead with the embodied-simulative account.

## Ethics statement

The study was conducted in line with the guidelines of the German Research Foundation—DFG, concerning ethical considerations of experimental studies involving human subjects. In particular, the current experiment is exempt from ethical approval procedures given that the EEG method is noninvasive and the material used in the experiment does not include any potentially harmful content. Thus, there are no particular risks related to this experiment and the experimental procedure.

## Author contributions

EC designed the research, carried out the experiment, processed the data, and analyzed the results. GB contributed to the discussion on the design. JK contributed to the analysis of the results. MW contributed to the design of the experiment. EC and MW wrote the paper.

### Conflict of interest statement

The authors declare that the research was conducted in the absence of any commercial or financial relationships that could be construed as a potential conflict of interest.

## Footnotes

^1^The controversy between Semantic Minimalism and Truth-conditional Pragmatics could be rephrased as a controversy about what should be understood as the meaning of a word. Following Frege's (1884, p. 71) famous quotation “One always has to take into account a complete sentence. Only therein do the words in effect have a meaning. […] It suffices if the sentence as a whole has a meaning, thereby also its parts obtain their meanings” (English translation cited after Werning, 2004), both parties agree that the meaning of a word is what it contributes to the meaning/intuitive truth-conditions of a sentence. Minimalists hold that this contribution is identical to the lexical entry or lexical meaning of a word, while Truth-conditional pragmatists want to make a distinction between the lexical entry of a word (what is stored in semantic long-term memory) and the meaning it contributes to the meaning/intuitive truth-conditions of the sentence. To account for the controversy, we reserve the expression “meaning of a word” for the contributory meaning and otherwise speak of lexical entry or lexical meaning of a word.

^2^The metaphysical status of affordances is being discussed in the philosophical literature. According to prominent views, affordances are properties of objects, however, dispositional and relational ones (cf. Chemero, 2003). They are mentally represented (e.g. involving canonical neurons), but are not representations themselves. One possibility discussed in this paper is that the attribution of a certain type of affordances to an object (i.e., *ad-hoc affordances*; see below for the distinction between *ad-hoc* and *generic affordances*) is the result of an online simulation of the situation linguistically described in the discourse context. In the generation of this simulation, world knowledge stored in the semantic memory might be relevant. When a subject lacks the relevant world knowledge, s/he might come to a different (or no) attribution of affordances to an object.

^3^Semantic Minimalism implies a two-step model on a naturalistic reading of this account. However, we do not want to implicate that supporters of this position (most notably, Emma Borg) necessarily endorse a naturalistic reading of Semantic Minimalism.

^4^The figure also shows a qualitative difference between the Telic combinations in the *Ad-hoc* affordance-inducing contexts (left red bar) vs. NonTelic combinations in Neutral contexts (right blue bar), with the N400 for the latter being more negative than the former. However, given our experimental design, these two conditions are statistically not comparable given that they differ with respect to both, the context and the noun-verb combination.

## Supplementary material

Supplementary Figure 1 **ERPs effects of Telic and NonTelic combinations presented in isolation**. Grand average waveforms measured on the verb for Telic and NonTelic combinations are shown for nine centro-parietal channels, together with the topography of the negativity effect (NonTelic condition minus Telic condition). The waveforms show a negativity effect for NonTelic noun-verb combinations compared to Telic combinations.

## Abbreviations

EEG: Electroencephalography
ERPs: Event-related brain potentials
N_CON: Neutral discourse context
A_CON: *ad-hoc* affordance-inducing context
N_CON-Telic: Telic combination in Neutral discourse context
N_CON-NonTelic: Nontelic combination in Neutral discourse context
A_CON-Telic: Telic combination in *ad-hoc* affordance-inducing context
A_CON-NonTelic: Nontelic combination in *ad-hoc* affordance-inducing context
TelicNV: Telic noun-verb combinations
NonTelic NV: Nontelic noun-verb combinations.
