# Supplementary material for: The Time-Course of Sentence Meaning Composition. N400 Effects of the Interaction between Context-Induced and Lexically Stored Affordances
Source: Front Psychol. 2017 May 26;8:813. doi: 10.3389/fpsyg.2017.00813 (PMC5445161; doi:10.3389/fpsyg.2017.00813)

Appendix A.
ERP effects of Telic and NonTelic noun-verb combinations presented in isolation

Given the relevance of the distinction between Telic noun-verb combination (TelicNV) and NonTelic noun-verb combination (NonTelicNV) in the logic of the present study, we carried out a second experiment to investigate the ERP effects of presenting those combinations in isolation. This experiment was conducted with the same participants who participated in the main study and after the completion of all other tasks, including the EEG experiment described in the main text.

Materials and Methods
The experimental 40 pairs of noun-verb combinations in the Telic condition and 40 pairs in the NonTelic condition were randomly intermixed with 25 incongruent filler pairs, and then distributed into two lists, such that each word could appear only once in each list. Each trial started with the presentation of a fixation cross in the center of the screen for 1300 ms followed by the first word of the pair (the noun), which was displayed for 400 ms, with 450-ms blank intervals before the presentation of the cue word (the verb), also for 400 ms. After an interval of 450 ms, a yes/no question was shown for 2000 ms asking the subjects whether the two words were semantically related or not, to which they responded by pressing a button. The questions were asked to make sure that the participants were alert during the task and did actually process the words, as confirmed by the accuracy of their answers that was well above chance. The ERPs were time-locked to the onset of the verb. Total time-on-task was approximately 5 minutes. The methods for the electroencephalogram recording and data processing were the same as in the EEG experiment described in the main text. In particular, a preliminary ANOVA including all EEG electrode sites was conducted, calculating average changes in amplitude over all channels per condition. Subsequently, a follow-up analysis was performed involving specifically a predetermined region over centro-parietal sites with the variables Context (2 levels: N_CON, A_CON), Combination (2 levels: Telic, NonTelic), and Electrodes (7 levels: CP1, CP2, CPz, Pz, P1, P2, POz). This region was selected because the N400 is known to be maximal over these sites and for consistency with the analyses conducted in the main experiment.

Results
One participant was excluded due to excessive artifacts. For the remaining 21 participants, average ERPs were computed over artifact-free trials per condition (average percentage of included trials = 98%, range = 80–100% across the two conditions). Figure 6 shows that NonTelic noun-verb combinations (NonTelicNV) elicited more negative ERPs than Telic noun-verb combinations (TelicNV) in the 400-500 ms time interval (this time interval was selected because in the main experiment we observed maximal N400 effects in this epoch). The difference was significant in an omnibus ANOVA using average amplitude per condition across all EEG electrodes, M(NonTelicNV) = −.45μV, M(TelicNV) = .51 μV, $t(20) = 2.547$, $p=.019$, CI .96 ± .79, and for a predetermined region over centro-parietal sites where the N400 is known to be maximal (CP1, CP2, CPz, Pz, P1, P2, POz; e.g., Kutas et al., 2006), M(NonTelicNV)= −.14 μV, M (TelicNV) = 1.13 μV, $t(20) = 3.036$, $p=.007$, CI 1.28 ± .88. As revealed by the topographical map, this negativity effect spreads to the frontal sites as well. We did not follow on this effect, though, because whereas it might be interesting in itself, it might be simply due to the specific experimental material used (single words rather than sentences) and its exploration fall beyond the scope of the present experiment.

Supplementary Figure 1

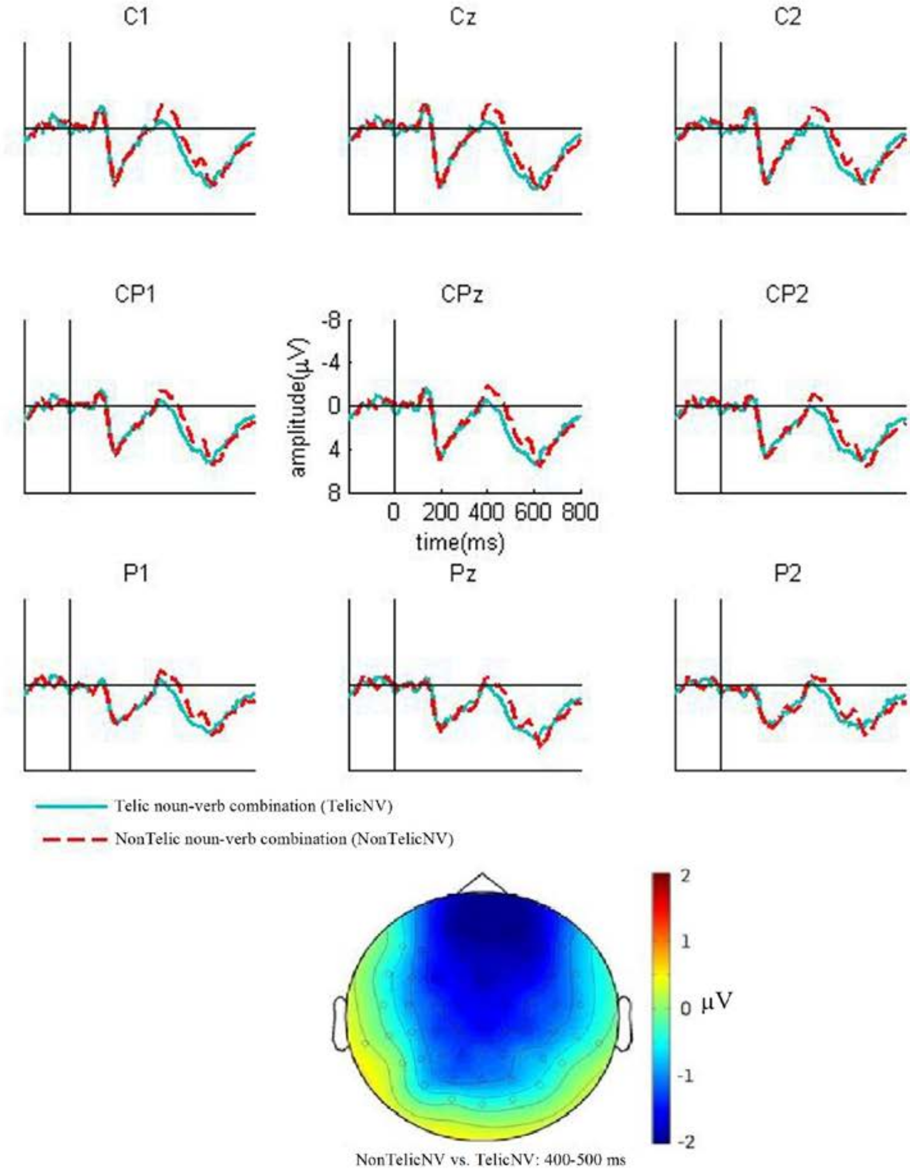

Supplement: Supplementary Figure 1 — ERPs effects of Telic and NonTelic combinations presented in isolation. Grand average waveforms measured on the verb for Telic and NonTelic combinations are shown for nine centro-parietal channels, together with the topography of the negativity effect (NonTelic condition minus Telic condition). The waveforms show a negativity effect for NonTelic noun-verb combinations compared to Telic combinations. [file Presentation1.pdf]
